# Switching from febuxostat to dotinurad, a novel selective urate reabsorption inhibitor, may substantially reduce serum urate levels, through URAT1 inhibition, potentially without adversely affecting ABCG2 function: findings from the SWITCH SURI study

**DOI:** 10.3389/fendo.2025.1655502

**Published:** 2025-10-02

**Authors:** Takeshi Osonoi, Shinichiro Shirabe, Miyoko Saito, Mitsuru Hosoya, Norie Watahiki, Satako Douguchi, Kensuke Ofuchi, Makoto Katoh

**Affiliations:** ^1^ Naka Kinen Clinic, Ibaraki, Japan; ^2^ Research Administration Center, Saitama Medical University, Saitama, Japan

**Keywords:** febuxostat, dotinurad, type 2 diabetic kidney disease, urate excretion transporter, serum urate, urate transporter 1 (URAT1), ABCG2, indoxyl sulfate

## Abstract

**Background:**

Dotinurad is a novel selective urate transporter 1 (URAT1) inhibitor recently introduced in Japan. This study aims to evaluate the efficacy and safety of dotinurad in hyperuricemic patients with type 2 diabetic kidney disease by comparing serum levels of urate and plasma and urinary levels of indoxyl sulfate excreted via the urate excretion transporter ATP binding cassette subfamily G member 2 (ABCG2), as indices, with baseline levels after switching from febuxostat to dotinurad in hyperuricemic patients with type 2 diabetic kidney disease (DKD).

**Methods:**

In this single-center, single-arm, open-label, prospective study, 37 hyperuricemic patients with DKD who had serum urate levels >6.0 mg/dL despite at least 3 months of febuxostat 20 mg/day were enrolled. Dotinurad was administered once daily for 24 weeks (starting at 0.5 mg/day, titrated up to 4 mg/day). The primary outcome was the proportion of patients achieving serum urate ≤6.0 mg/dL at week 24. Secondary outcomes included changes in serum urate, plasma and urinary indoxyl sulfate levels, and safety parameters.

**Results:**

At week 24, 70.3% of patients (26/37) achieved serum urate ≤6.0 mg/dL, significantly exceeding the predefined threshold of 30% (p < 0.01). Serum uric acid levels transiently increased from baseline 4 weeks after switching (p<0.01), but decreased to a mean of 5.5 mg/dL by 24 weeks (p<0.01). Urinary uric acid excretion increased significantly during the observation period, reflecting improved uric acid clearance. Urinary indoxyl sulfate (corrected for creatinine) showed significant increases at weeks 4 and 12, and indoxyl sulfate clearance was significantly improved at week 24. No clinically meaningful changes were observed in renal function, glycemic control, lipid profile, or blood pressure. Adverse events occurred in 17 patients (45.9%), mostly mild; two (5.4%) experienced serious events, with no drug-related safety concerns identified.

**Conclusion:**

Switching to dotinurad was significantly reduced serum urate levels via URAT1 inhibition, potentially without adversely affecting ABCG2 function, with the majority of patients achieving the target urate level (≤6.0 mg/dL) by week 24. Including the absence of new safety concerns, dotinurad appears to be an effective urate-lowering therapy for patients with type 2 DKD.

## 1 Introduction

The prevalence of gout has been steadily rising in Japan, with recent national health surveys estimating approximately 1.1 million affected individuals. Moreover, hyperuricemia—defined by elevated serum urate levels without necessarily manifesting as gout—affects over 10 million people nationwide (1). Beyond its established role in the pathogenesis of gout, hyperuricemia has emerged as a metabolic risk factor associated with a variety of lifestyle-related diseases, including hypertension, type 2 diabetes mellitus (T2DM), and atherosclerosis, even in asymptomatic individuals. Recent evidence also suggests a significant association between elevated serum urate and both the onset and progression of chronic kidney disease (CKD) (2–4). In particular, individuals with T2DM complicated by hyperuricemia face a markedly increased risk of CKD development compared to their normouricemic counterparts (5). Despite these observations, data regarding the contribution of hyperuricemia to CKD progression specifically in patients with diabetic kidney disease (DKD) remain limited.

Hyperuricemia is typically classified into three categories based on its pathophysiological mechanisms: reduced renal urate excretion, urate overproduction combined with diminished extrarenal clearance (renal overload), and a mixed type. Among these, reduced excretion is the most common, accounting for approximately 60% of cases, followed by the mixed (30%) and overload (10%) types (1). This distribution is thought to reflect, in part, the decline in glomerular filtration rate (GFR) seen in progressive CKD, which impairs renal urate elimination. Currently, urate-lowering therapies include xanthine oxidoreductase (XOR) inhibitors—such as febuxostat—which reduce urate production, and uricosuric agents that enhance renal urate excretion. In patients with renal dysfunction, XOR inhibitors are generally recommended regardless of hyperuricemia subtype (1, 6).

Renal handling of urate is mediated by a group of transporters, most notably urate transporter 1 (URAT1), which facilitates urate reabsorption in the proximal tubules. In contrast, urate excretion is primarily mediated by ATP binding cassette subfamily G member 2 (ABCG2), organic anion transporter (OAT) 1, and OAT3 (7, 8). Notably, ABCG2 is expressed not only in the kidney but also in the gastrointestinal tract and plays a critical role in urate and uremic toxin clearance, including that of indoxyl sulfate (9, 10). Accumulation of such toxins is associated with adverse cardiovascular outcomes and CKD progression (11). Importantly, both febuxostat and benzbromarone—an older uricosuric agent—have been shown to inhibit ABCG2-mediated urate transport (12), leading to increased plasma levels of its substrates in experimental models (13). These findings raise concerns about the potential for such agents to interfere with uremic toxin clearance, particularly in patients with compromised renal function.

Dotinurad is a recently developed selective urate reabsorption inhibitor (SURI) that exerts potent inhibitory effects on URAT1 while sparing ABCG2, OAT1, and OAT3 (14). This mechanism of action positions dotinurad as a potentially advantageous urate-lowering option in patients with CKD, minimizing interference with toxin excretion pathways. However, to date, no clinical studies have systematically evaluated the efficacy and safety of switching from an XOR inhibitor such as febuxostat to dotinurad in hyperuricemic patients with DKD.

The study protocol, including rationale and methods, has been previously published (15). The present article reports the clinical outcomes of that study.

## 2 Methods and analysis

### 2.1 Study design and patients

The SWITCH SURI study was a single-center, single-arm, open-label prospective trial of dotinurad in hyperuricemic patients with type 2 DKD. Adult patients (≥20 years) with type 2 diabetes and CKD (eGFR 30–60 mL/min/1.73m²) on stable febuxostat 20 mg/day for ≥3 months were eligible if their serum urate was >6.0 and <10.0 mg/dL before consent. Key exclusion criteria included an eGFR <30 mL/min/1.73m², acute gouty arthritis, liver dysfunction (aspartate aminotransferase [AST] or alanine aminotransferase [ALT] ≥100 IU/L), a history of urolithiasis, and concomitant use of medications affecting urate transporters (e.g., sodium-glucose cotransporter 2 [SGLT2] inhibitors, losartan, or irbesartan). All subjects provided written informed consent. The protocol was approved by the Japan Physicians Association Clinical Research Review Board (approval JPA007-2204-02). The study adhered to the Declaration of Helsinki and was registered in the Japan Registry of Clinical Trials (jRCTs031220080).

### 2.2 Intervention and assessments

Dotinurad will be started with URECE^®^ 0.5 mg tablet orally once daily within 1 week after the end of the baseline examination, and if the urate level remains above 6 mg/dL after ≥2 weeks, the dose of URECE^®^ tablets will be gradually increased (up to 4 mg), at observation points and at other scheduled visits, and continued until week 24 of the observation period. Study visits occurred at baseline and weeks 4, 12, and 24. Because of the potential to affect the evaluation of this study, the concomitant use of urate-lowering drugs (other than dotinurad), drugs that act on urate transporters (SGLT2 inhibitors, losartan, irbesartan), or drugs that affect indoxyl sulfate levels (spherical adsorptive carbon) is prohibited until the patient visit at 24 weeks after the start of dotinurad administration (except in case of discontinuation). However, in principle, drugs that patients already take before they provide consent may be continued from the date of consent until week 24, and the dosage and administration should be increased or decreased as appropriate according to medical conditions. Moreover, drugs deemed medically necessary may be added, except for the above prohibited concomitant medications. At each visit, serum and urinary urate and creatinine, plasma and urinary indoxyl sulfate, eGFR, albuminuria, and other safety labs were measured. Plasma and urinary concentrations of indoxyl sulfate were measured by liquid chromatography–tandem mass spectrometry (LC-MS/MS) at LSI Medience Corporation (Tokyo, Japan) (16). The detection limit of plasma and urinary indoxyl sulfate in this assay was 1.0 μg/mL. Values below the detection limit were calculated as half values of the detection limit. Urine was collected (spot sample) to assess urinary uric acid/creatinine and indoxyl sulfate/creatinine ratios. Uric acid clearance and indoxyl sulfate clearance were evaluated as the ratio to creatinine clearance. The primary efficacy endpoint was the percentage of patients achieving serum urate ≤6.0 mg/dL at week 24. Secondary endpoints included changes from baseline in serum urate, plasma and urinary indoxyl sulfate, and renal injury markers (eGFR, urinary albumin-to-creatinine ratio: UACR). Safety endpoints included incidence of adverse events (AEs), changes in liver enzymes, glucose metabolism, blood pressure, pulse rate (PR) and electrolytes, and oxidative stress.

### 2.3 Statistical analysis

Based on the study protocol, a one-sample proportion test (target rate >30%) with a two-sided significance level of α = 0.05 and 90% power required a sample size of 44 patients. The full analysis set (FAS) included all patients who received at least one dose of dotinurad and had at least one post-baseline efficacy assessment. The per-protocol set (PPS) comprised the FAS population excluding participants with major protocol deviations. For the primary efficacy endpoint—the proportion of patients achieving a serum urate level of ≤6.0 mg/dL at week 24—a one-sample proportion test was performed under the null hypothesis that the achievement rate equals 30%. In the FAS analysis, patients with missing serum urate values at week 24 were regarded as non-achievers. For secondary efficacy outcomes based on continuous variables, summary statistics were calculated at each observation point. Either a one-sample t-test or Wilcoxon signed-rank test was used, depending on the distribution, to assess within-group changes from baseline. Safety analyses included all patients who received at least one dose of dotinurad. When statistical tests were conducted, a two-sided p-value <0.05 was considered statistically significant.

## 3 Results

### 3.1 Patient disposition and baseline characteristics

**Figure 1:**
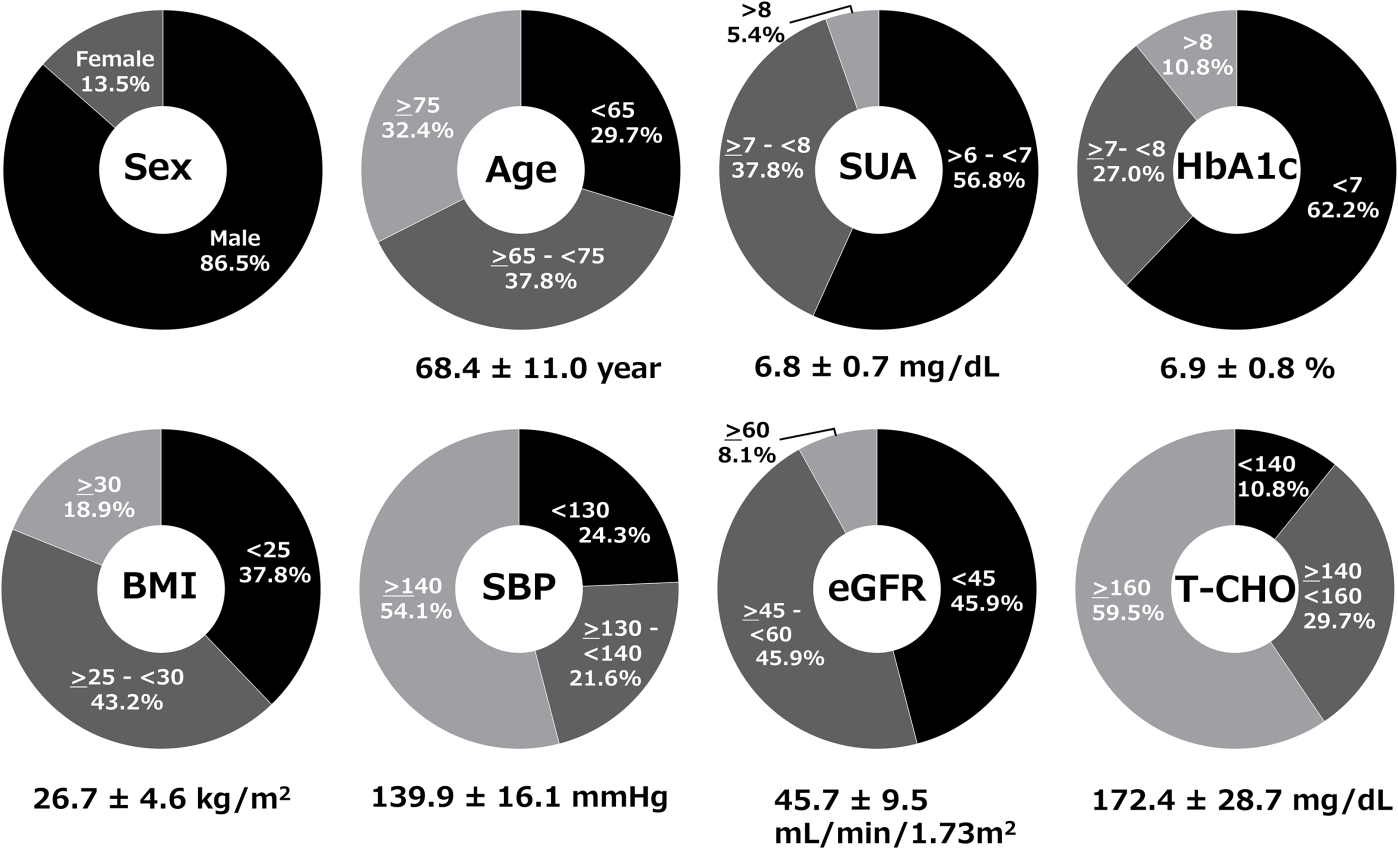
Baseline clinical characteristics of patients in the full analysis set (FAS). Demographic and clinical parameters of the 37 patients at baseline while receiving febuxostat 20 mg/day.

A total of 37 subjects gave their consent; 37 met inclusion criteria and entered the study. All 37 were included in the FAS and safety analysis. Two patients discontinued dotinurad (one due to a prohibited concomitant medication, and one patient did not attend any visits during the observation period); these remained in FAS but were excluded from the PPS (n=35). Baseline characteristics of the FAS (n=37) were: 86.5% male, mean age 68.4 ± 11.0 years, mean serum urate 6.8 ± 0.7 mg/dL, mean hemoglobin A1c (HbA1c) 6.9 ± 0.8%, mean body mass index 26.7 ± 4.6 kg/m^2^, mean systolic blood pressure (SBP) 139.9 ± 16.1 mmHg, mean eGFR 45.7 ± 9.5 mL/min/1.73m², and mean total cholesterol (T-CHO) 172.4 ± 28.7 mg/dL on febuxostat 20 mg/day (**Figure 1**).

The hyperuricemia patients with DKD in the study had hypertension (94.6%), dyslipidemia (91.1%), and cerebrovascular or cardiovascular disease (29.7%). The mean duration of hyperuricemia was 7.0 ± 5.6 years. At baseline, the following medications were prescribed for diabetes: α-glucosidase inhibitors (81.1%), metformin (67.6%), glucagon-like peptide-1 receptor agonists (GLP-1RAs) (35.1%), dipeptidyl peptidase-4 inhibitors (DPP4is) (29.7%), glinide (13.5%), and insulin (8.1%); for hypertension: angiotensin receptor blocker (ARB) (75.7%), calcium channel blockers (CCB) (67.6%), diuretic (32.4%) and β-blocker (16.2%); for dyslipidemia: 3-hydroxy-3-methylglutaryl-coenzyme A reductase inhibitor (statin) (48.6%), ezetimibe (32.4%) and fibrate (13.5%); for antiplatelet agents: 13.5%; and for anticoagulants: 8.1%.

### 3.2 Efficacy outcomes

**Figure 2:**
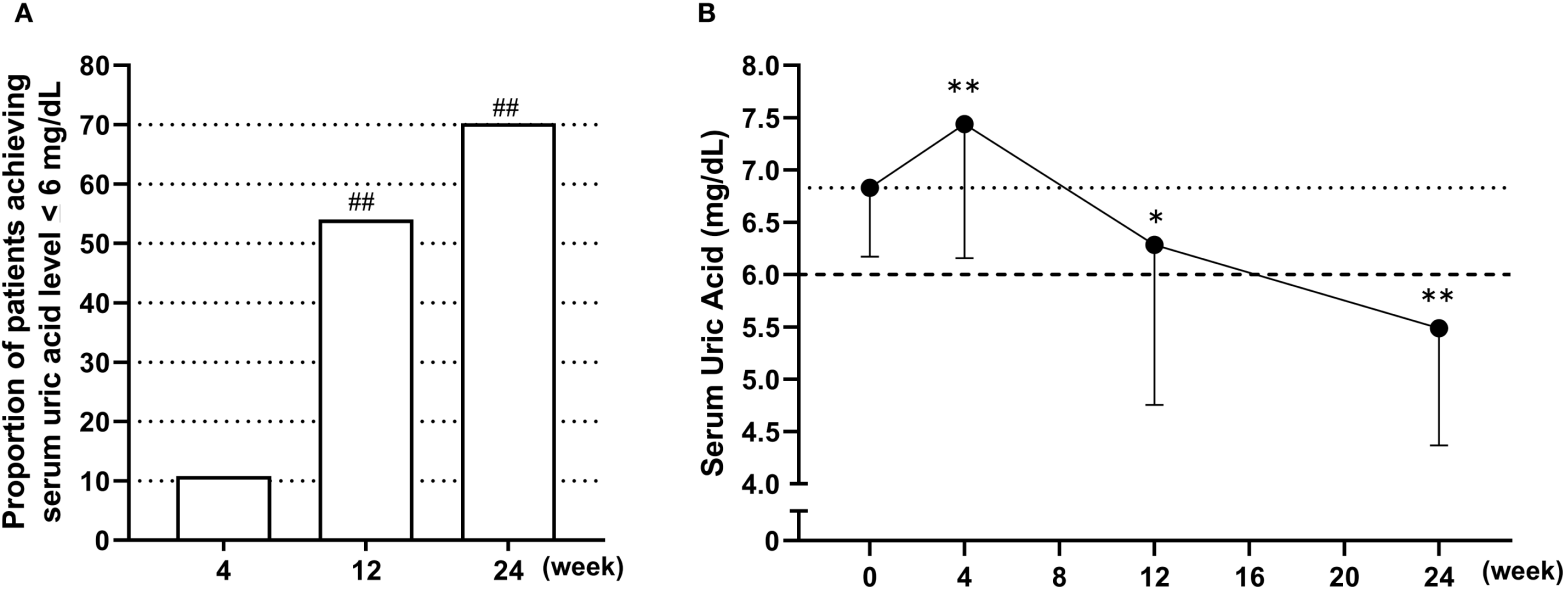
Achievement rates and time-course changes in serum urate levels following the switch from febuxostat to dotinurad. **(A)** Proportion of patients achieving serum urate levels ≤6.0 mg/dL at weeks 4, 12, and 24 following the switch from febuxostat to dotinurad. ^##^p < 0.01, based on a one-sample proportion test comparing the observed rate with the predefined threshold of 30%. **(B)** Time-course changes in mean serum urate levels from baseline to week 24 after switching to dotinurad. Data are presented as mean ± SD. *p < 0.05, **p < 0.01 vs. baseline (one-sample t-test).

**Figure 2** presents the achievement rates of serum urate levels ≤6.0 mg/dL (**Figure 2A**) and the changes in serum urate concentrations over time following the switch to dotinurad (**Figure 2B**). In the FAS, 26 of 37 patients (70.3%) achieved a serum urate level of ≤6.0 mg/dL at week 24. This rate substantially exceeded the predefined threshold of 30% (p < 0.01, one-sample proportion test). In the PPS, 26 of 35 patients (74.3%) also reached the target urate level. The achievement rates of serum urate ≤6.0 mg/dL were also observed at week 4 (10.8%) and week 12 (54.1%, P<0.01) (**Figure 2A**). After switching from febuxostat 20 mg/day to dotinurad 0.5 mg/day, a transient but significant increase in serum urate levels was observed at week 4. However, with dose escalation of dotinurad in accordance with the study protocol, serum urate levels significantly decreased by weeks 12 and 24 (**Figure 2B**).

**Figure 3:**
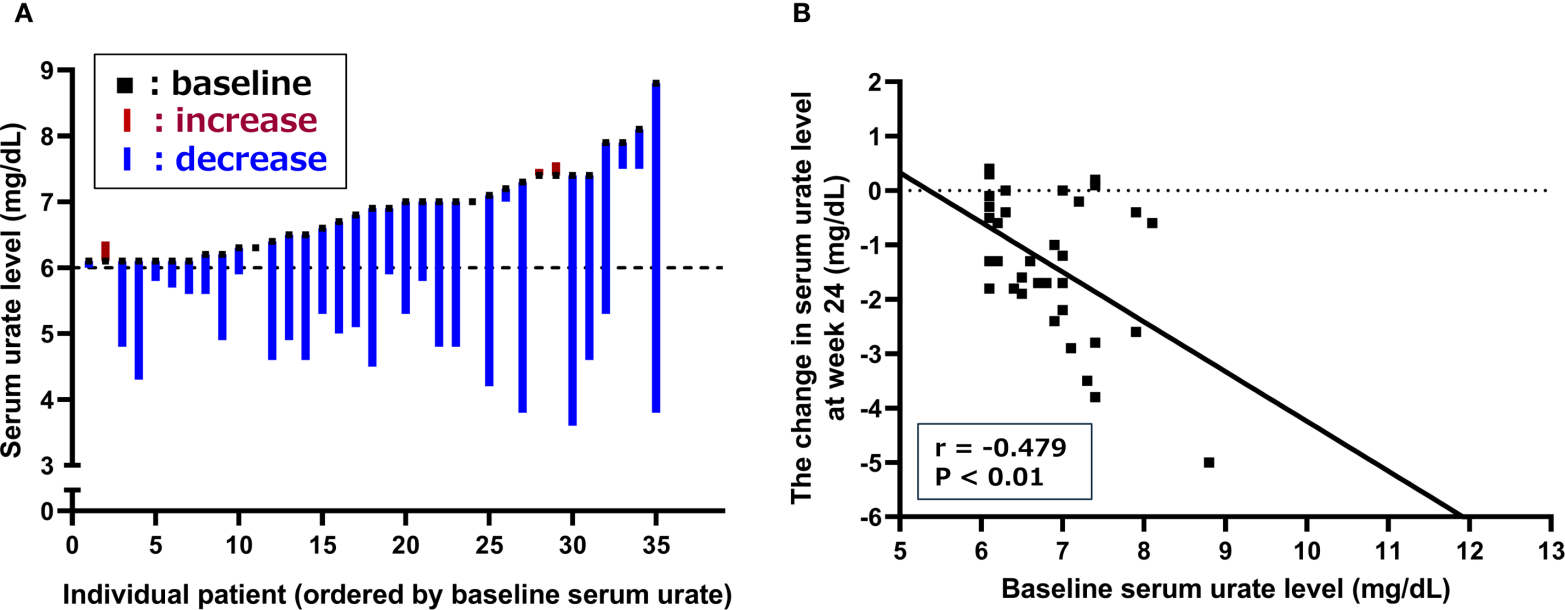
Changes in serum urate levels following the switch from febuxostat to dotinurad. **(A)** Individual changes in serum urate levels at week 24 for patients who completed the 24-week observation period, arranged in order of baseline serum urate levels. **(B)** Correlation between baseline serum urate levels and changes in serum urate at week 24 of dotinurad treatment.

**Figure 3A** illustrates the individual changes in serum urate levels at week 24 of dotinurad treatment for the 35 patients who completed the 24-week observation period, arranged in order of baseline serum urate levels. Of these, 30 patients (85.7%) showed a reduction in serum urate, 3 patients (8.6%) showed an increase, and 2 patients (5.7%) had no change. The correlation between baseline serum urate levels and the change in serum urate levels at week 24 was assessed and is shown in **Figure 3B**. A significant negative correlation was observed, indicating that higher baseline serum urate levels were associated with greater reductions at week 24 (r = -0.479, P < 0.01).

**Figure 4:**
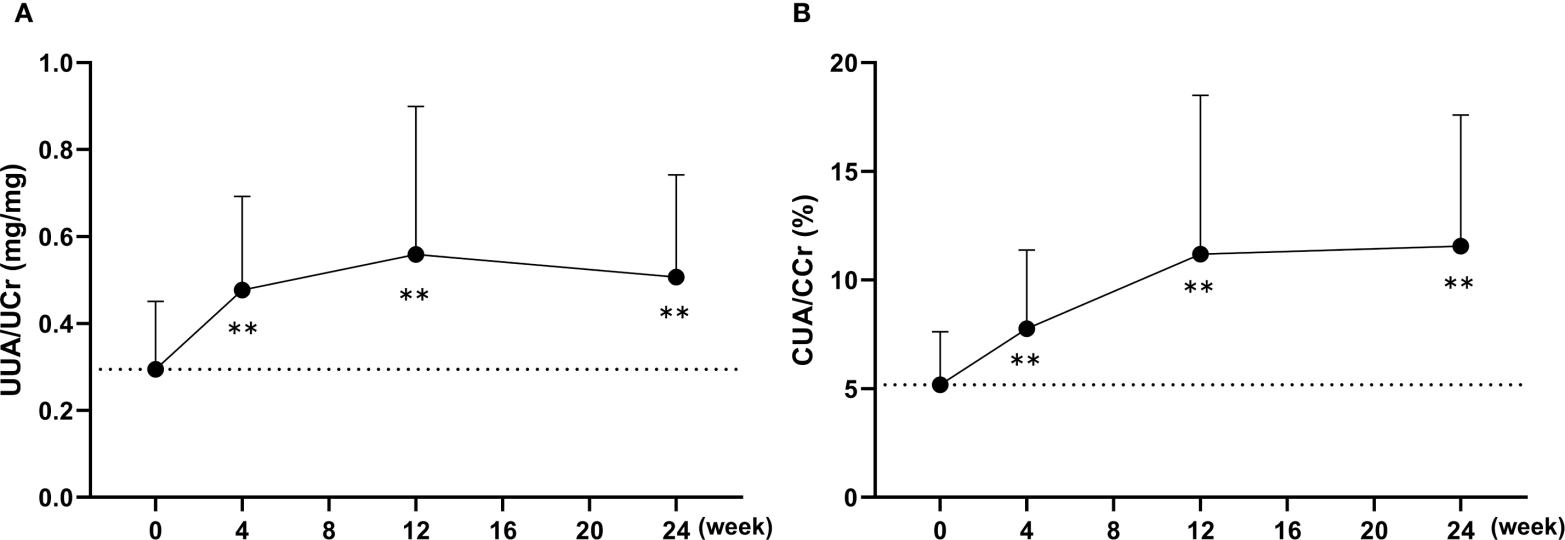
Changes in urinary uric acid excretion following the switch from febuxostat to dotinurad. **(A)** Time-course of the urinary uric acid-to-creatinine ratio (UUA/UCr) calculated from spot urine samples at each visit. **(B)** Changes in the ratio of uric acid clearance to creatinine clearance (CUA/CCr) over time after switching to dotinurad. Data are presented as mean ± SD. **p < 0.01 vs. baseline (one-sample t-test).

Using spot urine samples, urinary uric acid concentrations were corrected for urinary creatinine to calculate the urinary uric acid-to-creatinine ratio (UUA/UCr), and its changes over time are presented in **Figure 4A**. The UUA/UCr ratio showed a significant increase from baseline at week 4 and remained significantly elevated through week 24 following the switch to dotinurad. In addition, the ratio of uric acid clearance to creatinine clearance (CUA/CCr), which corresponds to fractional excretion of uric acid (FEUA), was calculated using serum and urinary concentrations of uric acid and creatinine, as shown in **Figure 4B**. The mean baseline CUA/CCr was 5.2 ± 2.4%, and this ratio also significantly increased from baseline at weeks 4, 12, and 24 after switching to dotinurad.

**Figure 5:**
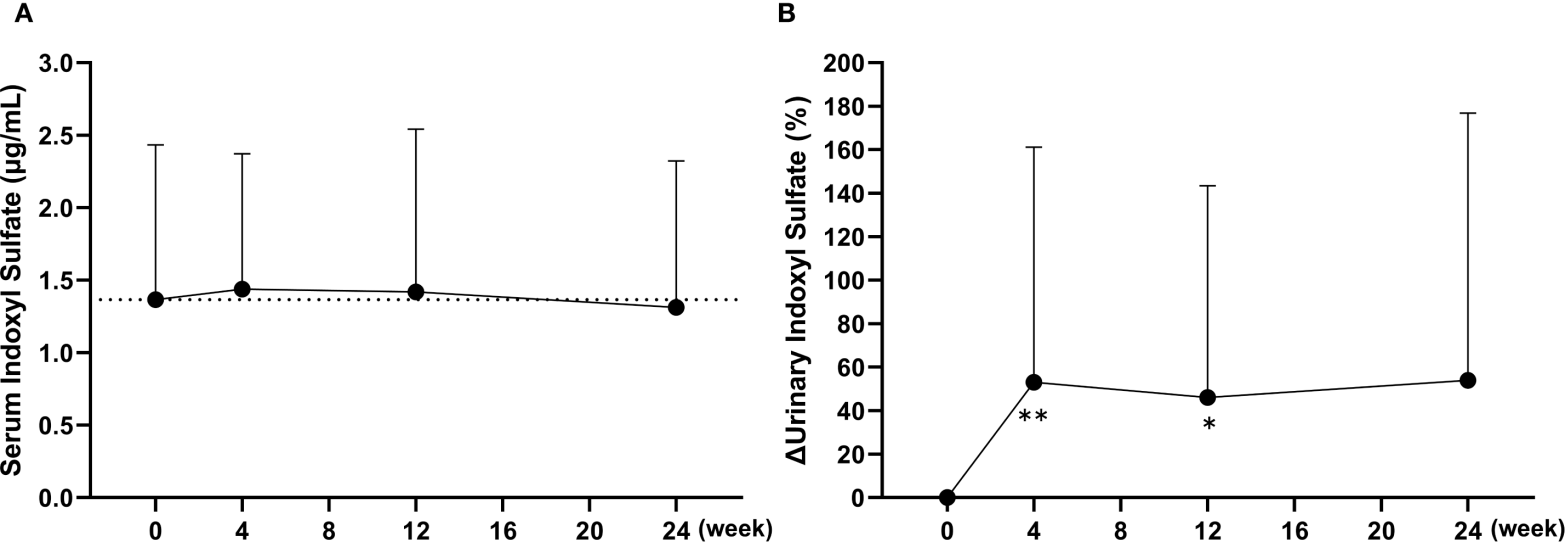
Changes in plasma and urinary concentrations of indoxyl sulfate following the switch from febuxostat to dotinurad. **(A)** Time-course of the plasma indoxyl sulfate concentrations at each visit. **(B)** Percent changes in urinary indoxyl sulfate excretion, adjusted for creatinine, over time after switching to dotinurad. Data are presented as mean ± SD. *p < 0.05, **p < 0.01 vs. baseline (Wilcoxon signed-rank test).

To evaluate the potential impact on ABCG2 function, plasma and urinary concentrations of indoxyl sulfate were measured. At baseline, plasma indoxyl sulfate levels were below the detection limit in approximately half of the participants (19 out of 37); therefore, no significant changes were observed in the overall cohort at any time point (**Figure 5A**). Consequently, a subgroup analysis was conducted in 15 patients with relatively high baseline levels (≥1.5 μg/mL). In this subgroup, mean plasma indoxyl sulfate concentrations demonstrated a decreasing trend from the baseline value of 2.5 ± 0.7 μg/mL, with values of 2.1 ± 0.9 μg/mL at week 4 (p < 0.01), 2.1 ± 1.3 μg/mL at week 12 (p = 0.07), and 2.0 ± 1.1 μg/mL at week 24 (p < 0.05). In contrast, urinary excretion of indoxyl sulfate, adjusted for creatinine, significantly increased at week 4 (p < 0.01) and week 12 (p < 0.05), suggesting a transient enhancement in renal elimination (**Figure 5B**). Notably, baseline urinary indoxyl sulfate levels were below the detection limit in only one participant (1 out of 37). Furthermore, the clearance ratio of indoxyl sulfate to creatinine (CIS/CCr), calculated from serum and urinary concentrations, gradually increased following the switch to dotinurad. This ratio rose from a baseline of 35.5 ± 15.6% to 46.2 ± 19.0% at week 24, representing a significant increase (p < 0.01).

eGFR (mL/min/1.73m²) showed a transient but significant increase at week 4 (47.4 Log UACR, P < 0.05) compared with baseline (45.7 ± 9.5), and remained slightly elevated at week 24 (46.6 ± 10.2), with no evidence of decline over the observation period. Log UACR did not show any significant changes throughout the study period (baseline: 2.0 ± 0.9, week 24: 1.8 ± 0.9).

**Figure 6:**
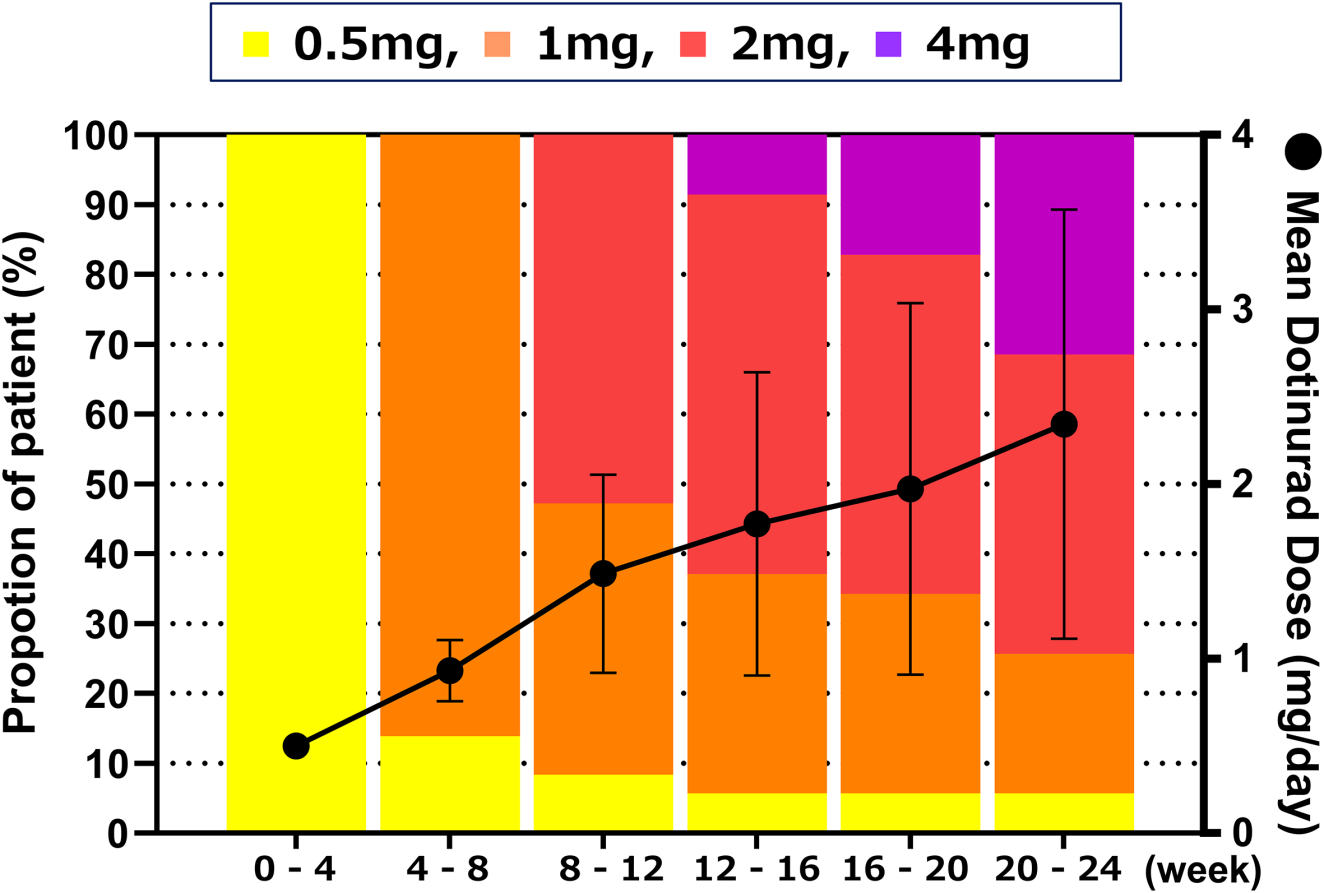
Distribution and mean daily dose of dotinurad over the 24-week treatment period following the switch from febuxostat to dotinurad. All patients initiated dotinurad at 0.5 mg/day, with dose titration based on serum urate levels according to the study protocol. Proportion of patients receiving each dose of dotinurad (left Y-axis, bar graph) and mean daily dose ± SD (right Y-axis, line graph) at each visit over the 24-week treatment period.

**Figure 6** shows the distribution and mean values of dotinurad doses over the 24-week treatment period following the switch. All patients initiated treatment with dotinurad at 0.5 mg/day, and the dose was gradually escalated based on individual serum urate levels in accordance with the study protocol. At week 24, the proportions of patients receiving 0.5, 1, 2, and 4 mg/day were 5.7%, 20.0%, 42.9%, and 31.4%, respectively, with a mean daily dose of 2.3 ± 1.2 mg.

### 3.3 Safety and tolerability

In this study, AEs occurred in 17 patients (45.9%), of whom 2 patients (5.4%) experienced serious AEs. Regarding disease-related events, no serious cases were reported, and non-serious conditions occurred in 3 patients (8.1%). The majority of these events were mild and transient in nature.

Among laboratory parameters related to liver enzymes, glucose metabolism, blood pressure, pulse rate, and electrolytes, a significant reduction in γ-glutamyl transpeptidase (γ-GTP) was observed after 24 weeks of dotinurad administration; no other significant changes were noted (**Table 1**).

**Table 1:** Effect of switching to dotinurad on laboratory parameters.

Parameter	N (baseline)	Baseline	24 weeks	Change (%)	*P*
AST(U/L)	37	27.8 ± 9.9	25.4 ± 12.9	-7.8 ± 28.4	0.11
ALT(U/L)	37	31.6 ± 22.4	29.0 ± 28.0	-9.8 ± 30.5	0.06
γ-GTP(U/L)	37	43.3 ± 27.9	37.0 ± 25.3	-8.0 ± 22.8	<0.05
HbA1c (%)	37	6.9 ± 0.8	6.9 ± 1.0	0.8 ± 8.8	0.59
BMI(kg/m^2^)	37	26.7 ± 4.6	26.5 ± 4.7	-0.9 ± 3.1	0.10
SBP(mmHg)	37	139.9 ± 16.1	134.9 ± 15.1	-2.5 ± 11.8	0.22
DBP(mmHg)	37	82.9 ± 10.4	80.4 ± 11.6	-2.7 ± 12.2	0.20
PR (beats/min)	37	80.3 ± 12.0	77.6 ± 13.1	-3.1 ± 12.4	0.14
Na(mEq/L)	33	142.1 ± 2.2	141.6 ± 2.6	-0.4 ± 1.6	0.19

Data are presented as mean ± standard deviation (SD). *P* values represent comparisons with baseline using one-sample t-tests. AST, aspartate aminotransferase; ALT, alanine aminotransferase; γ-GTP, gamma-glutamyl transferase; HbA1c, hemoglobin A1c; BMI, body mass index; SBP, systolic blood pressure; DBP, diastolic blood pressure; PR, pulse rate; Na, serum sodium.

Furthermore, to evaluate the effect of switching to dotinurad on oxidative stress, serum high-sensitivity C-reactive protein (hs-CRP) and urinary 8-hydroxy-2’-deoxyguanosine (8-OHdG) levels were assessed. Serum hs-CRP showed no significant changes at week 24 (0.14 ± 0.23 mg/mL) compared with baseline (0.12 ± 0.14 mg/mL). In contrast, urinary 8-OHdG, a marker of oxidative DNA damage, significantly decreased from a baseline value of 10.0 ± 3.0 ng/mgCre to 8.4 ± 3.4 ng/mgCre at week 4 (p < 0.01) and 8.6 ± 3.6 ng/mgCre at week 24 (p < 0.05).

## 4 Discussion

In this study, we evaluated the efficacy and safety of switching from febuxostat to dotinurad in hyperuricemic patients with type 2 DKD. Dotinurad was administered once daily at doses ranging from 0.5 mg to a maximum of 4 mg for 24 weeks. The results demonstrated that dotinurad effectively reduced serum urate levels to the target threshold of ≤6 mg/dL, and no major safety concerns were identified during the treatment period. These findings suggest that dotinurad is an effective uricosuric agent for serum urate control and may serve as a convenient and promising therapeutic option for hyperuricemia in patients with DKD.

The efficacy of switching from febuxostat to dotinurad in patients with hyperuricemia, as well as the effectiveness of dotinurad in patients with type 2 DKD, has not yet been sufficiently investigated in clinical practice. In the present study, dotinurad demonstrated a gradual increase in the rate of serum urate reduction and achievement of the target level of ≤6.0 mg/dL from week 4 onward, appearing to stabilize by week 24, with achievement rates of 70.3% in the FAS and 74.3% in the PPS. A separate study evaluating the efficacy of dotinurad in patients with CKD reported that, with similar dose titration, 72% of patients in CKD stages G3/G4 achieved the target urate level at week 24, which was non-inferior to the achievement rate in the G1/G2 group (17). Additionally, a recent retrospective study reported that, among eight CKD patients who were switched from febuxostat to dotinurad, 63% were able to achieve or maintain serum urate levels below 6.0 mg/dL (18). These findings are consistent with the results of the current study and suggest that dotinurad maintains its therapeutic efficacy even in patients with renal impairment who exhibit insufficient response to febuxostat.

**Figure 7:**
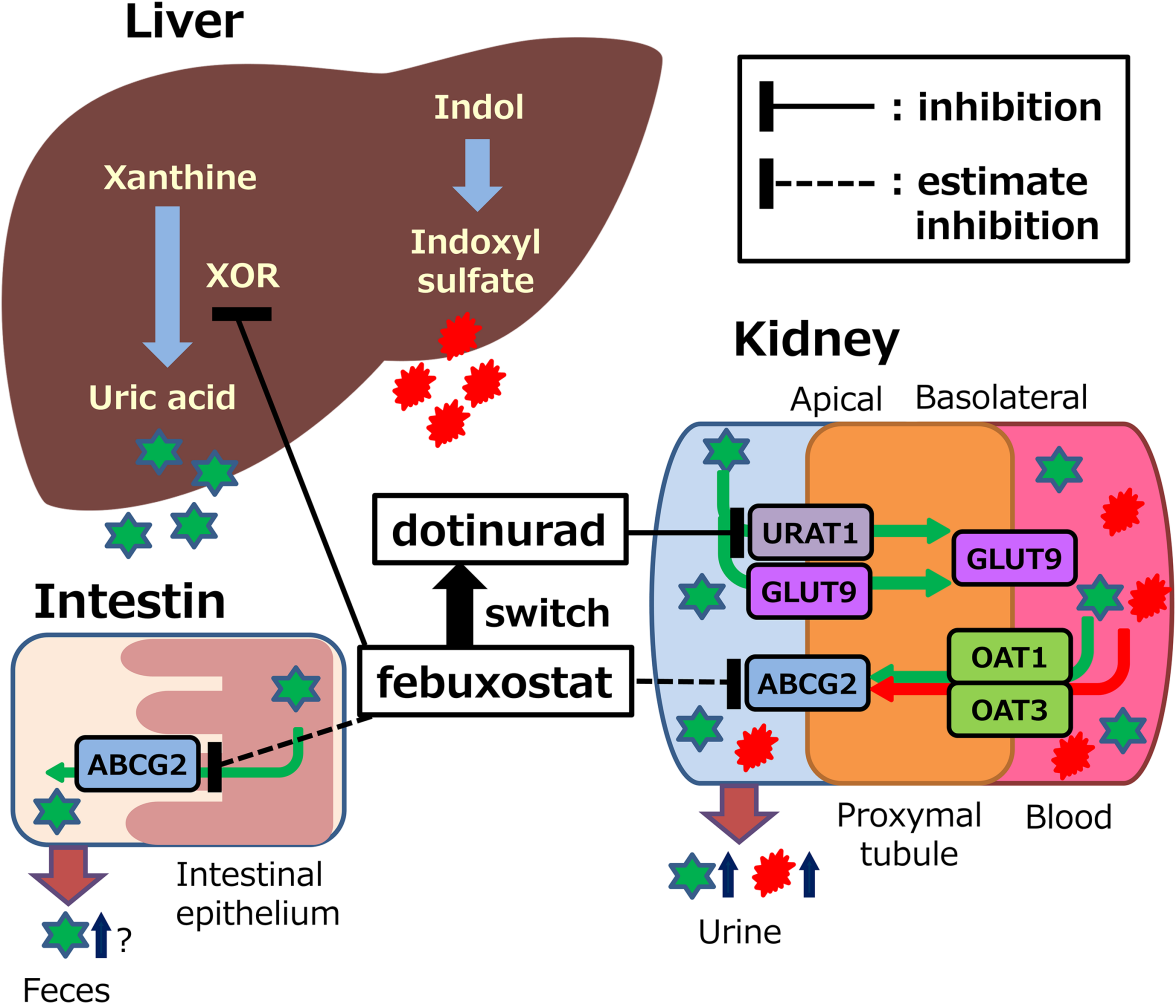
Potential mechanisms of action of febuxostat or dotinurad on synthesis and excretion of uric acid and indoxyl sulfate. XOR, xanthine oxidoreductase; URAT1, urate transporter 1; ABCG2, ATP binding cassette, subfamily G, 2; OAT, organic anion transporter; GLUT9, glucose transporter 9. This figure was adapted with minor modifications from our published study protocol (15).

In this study, ABCG2 genotyping, measurements of other uremic toxins, and 24-hour urine collection were not performed; therefore, a direct assessment of ABCG2 function was not possible. Nevertheless, nonclinical studies have reported that febuxostat inhibits the urate excretion transporters ABCG2, OAT1, and OAT3 (12). These transporters are involved in the renal excretion of both urate and indoxyl sulfate (10). Thus, febuxostat may inhibit the excretion of urate and indoxyl sulfate (**Figure 7**). Febuxostat was shown to inhibit ABCG2 in overexpressing cell systems at clinically relevant concentrations (maximum plasma concentration approximately 1.7 μmol/L at 20mg tablet), with an IC50 value of 0.027 μmol/L (12). In contrast, dotinurad (maximum plasma concentration of approximately 0.28 μmol/L at a 1-mg dose) inhibited urate transport in a concentration-dependent manner in cells overexpressing URAT1 and ABCG2, with IC_50_ values of 0.0372 μmol/L and 4.16 μmol/L, respectively (13). The ratio of IC_50_ values was 112, indicating that dotinurad has markedly lower inhibitory activity against ABCG2 compared with URAT1, and that at clinically relevant concentrations it does not reach the IC_50_ value for ABCG2. In this way, dotinurad has been shown to exert selective and potent URAT1 inhibitory activity, with minimal effects on ABCG2, OAT1, and OAT3 (13, 14). However, indirect or direct evaluation in clinical settings has not yet been conducted.

In the FAS, plasma indoxyl sulfate concentrations did not change significantly following the switch to dotinurad. However, approximately half of the participants had baseline plasma indoxyl sulfate levels below 1.0 μg/mL. A previous report in patients with coronary artery disease demonstrated that plasma indoxyl sulfate concentrations tend to increase as eGFR declines, yet even in patients with eGFR ranging from 30 to 59 mL/min/1.73m², the median plasma indoxyl sulfate level was 1.0 μg/mL, with an interquartile range of 0.71–1.5 μg/mL (19). This observation is consistent with our findings and suggests that a higher baseline concentration of indoxyl sulfate may be necessary to adequately evaluate the pharmacological effects of dotinurad on urate transporters. Accordingly, a subgroup analysis was conducted in 15 patients with baseline plasma indoxyl sulfate levels ≥1.5 μg/mL. In this subgroup, plasma indoxyl sulfate levels significantly decreased following the switch to dotinurad. Furthermore, urinary excretion of indoxyl sulfate, adjusted for creatinine, showed a significant increase. These findings suggest that switching to dotinurad may enhance the function of transporters involved in the excretion of uremic toxins, such as ABCG2.

Urate is primarily synthesized in the liver, with approximately 70% excreted via the kidneys and the remaining 30% via the small intestine (20, 21). Intestinal urate excretion is specifically mediated by ABCG2 (9, 22). These findings suggest that dotinurad may facilitate urate excretion not only through URAT1 inhibition but also via ABCG2-mediated pathways. Given that ABCG2 promotes urate excretion through both the kidneys and the intestines, it may contribute to the urate-lowering effects of dotinurad (**Figure 7**). This dual mechanism may partly explain the high urate-lowering rates observed in this and other long-term studies, such as a phase 3 trial showing achievement rates of 91.3% with 2 mg/day and 100% with 4 mg/day over 58 weeks (23).

Additionally, in patients with impaired renal impairment, the level of indoxyl sulfate in the circulating blood increases, thus a risk factor for CKD progression and cardiovascular complications due to its accumulation in the blood and various organs (11). Thus, lowering plasma indoxyl sulfate concentrations with dotinurad may be associated with a lower risk of cardiovascular disease development and renal disease progression than febuxostat. However, further studies are needed for the development of cardiovascular disease, which requires long-term observation periods in large clinical studies.

It is known that URAT1 inhibitors may be associated with high incidence of serum creatinine elevations and renal-related AEs (24). The mechanism underlying the elevation of sCr levels is not fully known, but it may be due to increased excretion and microcrystallization of urinary uric acid in renal tubules. A long-term phase 3 study assessing the safety of dotinurad monotherapy demonstrated no noteworthy changes with respect to renal impairment and renal parameters (23). In addition, renal function had significantly improved from the baseline with long-term treatment on dotinurad (23). In this study, dotinurad did not adversely affect baseline eGFR or UACR in patients with DKD. However, given the relatively short 24-week treatment period, longer-term data may be necessary to fully elucidate the potential renal benefits of dotinurad.

Febuxostat 20 mg/day was selected based on the highest percentage of the dose prescribed in real-world database using the JMDC Epidemiology Receipt Database in Japan (25). Furthermore, the cumulative achievement rates of febuxostat for serum urate levels of ≤6 mg/dL in patients with gout or asymptomatic hyperuricemia (25) or with hyperuricemia and CKD (26) were 51% and 41% at 20 mg/day and 60% and 53% at 40 mg/day, respectively, with no significant increase in the efficacy for an increase to the maintenance dose. In addition, febuxostat may be hesitant to increase the dose because the CARES trial, received at an initial dose of 40 mg/day febuxostat, showed a higher incidence of CV events than allopurinol (27). Therefore, 20 mg/day was considered the most clinically relevant comparator for evaluating the effect of switching to dotinurad. Hyperuricemia should be recognized as a cardiovascular risk factor, although it remains uncertain whether it represents an independent causal factor or merely a marker (28). The potential benefit of urate-lowering therapy on cardiovascular outcomes also remains under debate, and individualized treatment strategies are recommended (28). In a phase 2 study investigating the dose-dependent urate-lowering effects of dotinurad in hyperuricemic patients with or without gout, including elderly individuals and those with renal impairment, the proportion of patients achieving serum urate levels ≤6.0 mg/dL after 12 weeks of treatment was 23.1% (9/39) with 0.5 mg/day, 65.9% (27/41) with 1 mg/day, 74.4% (29/39) with 2 mg/day, and 100% (40/40) with 4 mg/day; no patients in the placebo group (0/39) achieved the target (14).

In the present study, a transient increase in serum urate levels was observed at week 4 following the switch to dotinurad, which is likely attributable to the insufficient efficacy of the initial 0.5 mg/day dose. Previous studies have reported that the urate-lowering effect of febuxostat 20 mg/day is non-inferior to that of dotinurad 1 mg/day (29), suggesting that the initial dose of 0.5 mg/day used in this study was insufficient to achieve adequate urate reduction. Subsequent dose escalation in accordance with the study protocol resulted in a rapid reduction in serum urate levels. Although no cases of gout flares or deterioration in clinical parameters were observed during this transient elevation, caution may be warranted regarding the potential for urate rebound when switching from febuxostat 20 mg/day to dotinurad 0.5 mg/day.

A recent prospective, randomized, open-label trial comparing the urate-lowering effects of the uricosuric agent benzbromarone (25 mg/day) and the xanthine oxidoreductase (XOR) inhibitor febuxostat (20 mg/day) in male patients with gout and reduced renal urate excretion demonstrated that the proportion of patients achieving the target serum urate level of <6 mg/dL after 12 weeks was significantly higher in the benzbromarone group than in the febuxostat group (30). Since benzbromarone, like febuxostat, inhibits ABCG2-mediated urate transport (12), these findings suggest that URAT1 inhibitors may exert greater urate-lowering efficacy than XOR inhibitors in patients with urate underexcretion-type hyperuricemia. In a simplified classification method using spot urine samples, a urinary urate-to-creatinine ratio (UUA/UCr) ≥0.8 indicates urate overproduction type, while ≤0.4 indicates underexcretion type (31). It is generally accepted that the majority of patients with hyperuricemia fall into the underexcretion category (1). Although all patients in the present study were evaluated while receiving febuxostat, which may obscure precise classification, none had a UUA/UCr ratio ≥0.8, and 31 of 37 patients (83.8%) had values ≤0.4, suggesting that most cases were likely of the urate underexcretion type. Therefore, switching to dotinurad in patients with an insufficient response to febuxostat may represent a rational treatment approach based on pathophysiological classification.

In Japan, urate-lowering drugs are approved for the treatment of both asymptomatic hyperuricemia and gout. The treatment target of SUA ≤6.0 mg/dL adopted in this study was derived from both domestic and international guidelines (1, 32), with the primary rationale of preventing urate crystal formation and recurrent gout flares. All participants in this study had asymptomatic hyperuricemia, and the study was not designed to evaluate the effects of SUA lowering on CKD progression. Accordingly, the findings should be interpreted as exploratory pharmacological observations rather than direct evidence of renal outcome modification.

This study has several limitations. First, the sample size was small (n=37), which inevitably limits the statistical power and the precision of effect estimates. Second, as this was a single-center, open-label, single-arm study without a control group conducted in Japan, the participants were predominantly of a single ethnic background, thereby limiting the external validity of the findings. Third, although the underrepresentation of women in clinical trials of urate-lowering therapies has been identified as a significant issue (33), the female participation rate in the present study remained low at 13.5%. Nevertheless, because all consecutive eligible patients were enrolled without regard to sex, this proportion is comparable to, or even higher than, that reported in previous trials evaluating urate-lowering therapies in asymptomatic hyperuricemic populations (33). Finally, the relatively short follow-up period (24 weeks) precluded a robust assessment of long-term cardiovascular and renal outcomes. Despite these limitations, this is the first study to demonstrate that switching to dotinurad reduces plasma indoxyl sulfate and increases its urinary excretion, suggesting minimal inhibitory effects on uremic toxin transporters such as ABCG2, OAT1, and OAT3. While caution is required when extrapolating these results, the present study provides important preliminary evidence that warrants validation in larger, multi-ethnic, and more gender-balanced populations with longer follow-up.

## 5 Conclusion

Uricosuric agents have traditionally been considered less effective in patients with impaired renal function due to their mechanism of action, and their use in patients with CKD has been limited. However, in the present study, dotinurad achieved a target serum urate level in over 70% of patients with DKD, demonstrating favorable efficacy even in this population. In particular, patients who exhibited an insufficient response to febuxostat may have had underexcretion-type hyperuricemia, highlighting the potential utility of selecting urate-lowering agents based on pathophysiological classification. Moreover, the findings suggest that dotinurad may promote urate excretion not only via URAT1 inhibition but also through ABCG2-mediated pathways. Taken together, these results indicate that dotinurad is an effective uricosuric agent for serum urate control and may serve as a convenient and promising treatment option for hyperuricemic patients with DKD, especially those with insufficient response to XOR inhibitors.

## Data Availability

The datasets presented in this article are not readily available because The data supporting the findings of this study are available from the corresponding author upon reasonable request. Due to ethical restrictions and participant confidentiality, access to the data may be limited and will be provided in accordance with institutional guidelines and applicable data-sharing policies. Requests to access the datasets should be directed to m_katou@saitama-med.ac.jp.
